# Advances in research on malignant transformation of endometriosis-associated ovarian cancer

**DOI:** 10.3389/fonc.2024.1475231

**Published:** 2024-10-09

**Authors:** Fang Chen, Mengying Zhu, Wenjuan Li

**Affiliations:** ^1^ Department of Gynecology, People’s Hospital of Liaoning Province, Shenyang, China; ^2^ Liaoning University of Traditional Chinese Medicine, Shenyang, China

**Keywords:** endometriosis, endometriosis-associated ovarian carcinoma, malignant transformation, ovarian clear cell carcinoma, ovarian endometrioid carcinoma

## Abstract

Endometriosis (EMs) is a prevalent chronic gynecological condition that depends on estrogen, marked by the presence of active endometrial tissue (glands and stroma) outside the uterus. Although pathologically benign, it exhibits biological behaviors such as invasion and metastasis akin to malignant tumors. Endometriosis-associated ovarian carcinoma (EAOC), arising from malignant transformation of EMs, poses significant clinical challenges. However, the mechanisms underlying EAOC pathogenesis remain incompletely understood, with a lack of reliable biomarkers for early diagnosis and personalized treatment strategies. Considering the significant number of EMs patients and the extended period during which malignant transformation can occur, EAOC deserves significant attention. Current research both domestically and internationally indicates that the pathogenesis of EAOC is complex, involving genetic mutations, immune microenvironment, oxidative stress, epigenetic changes, and related areas. This review summarizes the mechanisms underlying the development of EAOC.

## 1 Introduction

EMs is a chronic inflammatory disease characterized by the presence of ectopic lesions, most commonly in the ovaries, and presenting with symptoms such as pelvic pain, dysmenorrhea, and infertility (1). It affects 10-18% of women of reproductive age worldwide (2). Despite its benign nature, EMs exhibits biological features akin to malignancy, including histological infiltration and angiogenesis. EAOC, resulting from malignant transformation of EMs, refers to specific types of ovarian cancer (3). Epidemiological studies indicate that EMs increases the risk of developing certain histological subtypes of ovarian cancer, predominantly ovarian clear cell carcinoma (OCCC) and ovarian endometrioid carcinoma (OEC) (4). The lifetime risk of ovarian cancer in EMs patients is modest (~1.9%), yet higher compared to the general female population (~1.4%) (5). Furthermore, clinical diagnosis is complicated by rapid tumor growth that may obscure benign tissues, and challenges in pathological sampling which can lead to underdiagnosis of coexisting benign or atypical EMs lesions in EAOC patients (6). This underscores the difficulty in studying EAOC comprehensively. Early detection of malignant transformation of EMs and identification of early events are crucial for improving prognosis in EAOC. Therefore, understanding the mechanisms and biomarkers associated with EMs malignant transformation is essential. This review discusses current insights into the mechanisms underlying EAOC development, and outlines future directions for basic research and clinical translation, aiming towards stratified management and personalized treatment of ovarian cancer patients.

## 2 Malignant transformation mechanism

### 2.1 Genetic mutations

AT-Rich Interaction Domain 1A (ARID1A), a tumor suppressor gene encoding the BAF Family Member 250a(BAF250a) protein, is a critical component of the SWI/SNF chromatin remodeling complex. It is the most commonly mutated gene within the SWI/SNF complex, and its loss-of-function mutations play a crucial role in dysregulating the expression of oncogenic and tumor suppressor genes, particularly in the context of EAOC (7). Genetic sequencing studies have revealed ARID1A mutations in 46% of OCCC patients and 30% of OEC patients. Previous research has shown that ARID1A mutations promote abnormal activation of the PI3K-Akt signaling pathway and interact synergistically with zinc-finger protein 217 (ZNF217), which may contribute to OCCC development (8). The PI3K/Akt signaling pathway is a pivotal pathway involved in tumor cell proliferation, invasion, and metastasis (9). Guan et al. demonstrated that ARID1A, functioning as a tumor suppressor gene, interacts with the p53 protein to inhibit cell proliferation through p53-dependent transcriptional regulation factors. Mutations in either Tumor Protein P53 or ARID1A can disrupt tumor-suppressive transcription, leading to uncontrolled cell proliferation and ultimately promoting EAOC development (10).

Phosphatase And Tensin Homolog (PTEN), located on chromosome 10, is a tumor suppressor gene involved in cell regulation, inhibiting tumor cell proliferation, adhesion, migration, and angiogenesis (11). Mutations in PTEN enhance the anti-apoptotic capability of endometrial cells, disrupt cell cycle regulation mechanisms, and promote retrograde implantation and survival of endometrial tissue (12). Another study indicates that low PTEN expression increases the invasive capacity of endometrial cells, facilitating the growth of ectopic endometrial tissue (13). Research by Dinulescu et al. demonstrates that ovarian surface epithelium-specific oncogenic K-ras or conditional PTEN loss of expression can lead to atypical hyperplasia resembling endometrioid adenocarcinoma structures, suggesting mutations and expression loss may be early drivers of OEC development (14).

Phosphatidylinositol-4,5-Bisphosphate 3-Kinase Catalytic Subunit Alpha (PIK3CA), a member of the PI3K-AKT-mTOR signaling pathway, is closely implicated in OCCC pathogenesis through its mutations (15). Activation of PIK3CA promotes expression of downstream genes in the PI3K-Akt pathway, inhibiting tumor cell apoptosis and facilitating tumor initiation and progression (16). Furthermore, PIK3CA mutations enhance PI3K activity, promoting cell cycle progression, proliferation, and migration (17). Co-mutation of PIK3CA and ARID1A enhances sustained IL-6 production, driving rapid tumor growth (18). A study by Chandler et al. in mice demonstrated that concurrent activation of mutated ARID1A and PIK3CA genes induces OCCC development (19).

**Figure 1:**
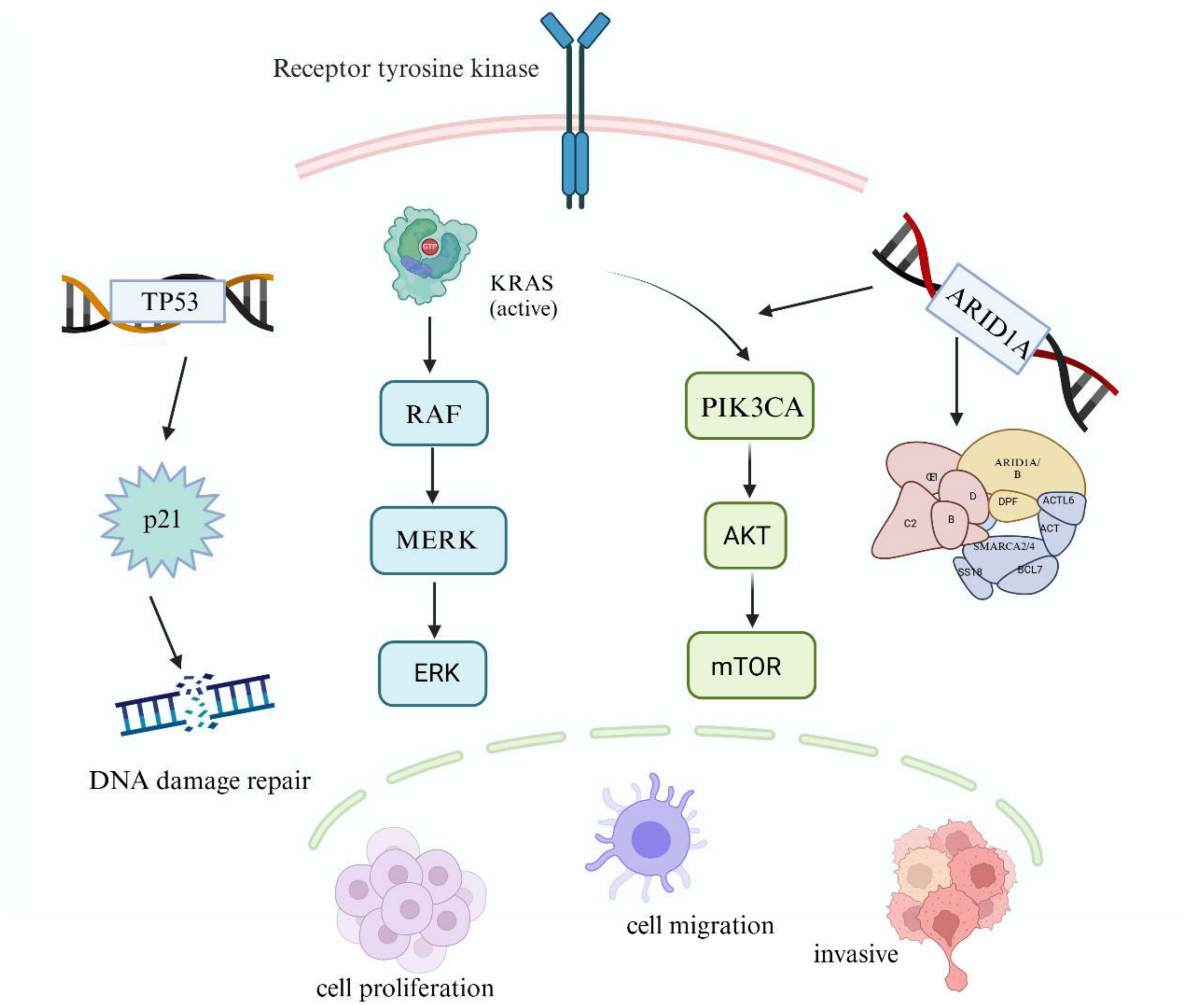
Mechanisms of gene mutations in EAOC.

The KRAS Proto-Oncogene (KRAS) is a major subtype of RAS mutations, encoding a signaling protein that can be activated by various extracellular stimuli and transduce signals through the downstream RAS/MAPK signaling pathway, promoting malignant biological behaviors such as cell proliferation, invasion, and migration (20, 21). Studies have found that 42.6% of EMs cases exhibit somatic KRAS mutations (22). Somatic mutations in KRAS may represent critical driver events during the malignant transformation of EMs (20). To date, all KRAS mutations detected in EMs have been located within “hotspot” sequences, indicating biological significance of KRAS hotspot mutations in EMs. DNA sequencing results suggest that KRAS mutations may represent a late event in the malignant progression of EMs, associated with OCCC occurrence (23). Overexpression of oncogenic KRAS in ovarian surface epithelium or conditional PTEN loss can lead to precancerous changes in endometrial glandular morphology; their concurrent presence can induce the development of OEC (24) (**Figure 1**).

The KRAS Proto-Oncogene (KRAS), a major mutant subtype of RAS, encodes a signaling protein activated by various extracellular stimuli, which transmits signals to the downstream RAS/MAPK signaling pathway. The tumor suppressor gene P53 (TP53) mutations impact the expression of its transcription-dependent tumor suppressor genes. Phosphatidylinositol-4,5-Bisphosphate 3-Kinase Catalytic Subunit Alpha (PIK3CA) activation enhances the expression of downstream PI3K-Akt pathway genes, inhibiting apoptosis and promoting tumor development. Additionally, AT-Rich Interaction Domain 1A (ARID1A), a frequently mutated gene in the SWItch/Sucrose Non-Fermentable(SWI/SNF) complex, leads to abnormal activation of the PI3K-Akt signaling pathway. Gene mutations play a critical role in the malignant transformation of endometrial cells by affecting DNA damage repair, cell proliferation, and invasion. (Created with BioRender.com).

### 2.2 Steroid hormone action

As is widely recognized, EMs is an estrogen-dependent disease, with estrogen and progesterone playing crucial roles in maintaining normal uterine endometrial function. whereas in ectopic endometrial tissue compared to normal endometrium, mRNA levels of the Cytochrome P450 Family 1 Subfamily B Member 1(CYP1B1) gene are significantly elevated (25). CYP1B1 participates in estrogen metabolism within the ectopic endometrial microenvironment, converting androgens such as androstenedione or testosterone into estriol and estradiol (25). High concentrations of estrogens can exert direct toxic effects on DNA of ectopic endometrial cells. Additionally, estrogens play critical roles in cell survival, proliferation, and inflammatory responses (26). Substantial research suggests that dysregulations in estrogen receptor (ER) and progesterone receptor (PR) expression are linked to the malignant progression of endometriosis (27). Exposure to estrogens, such as via hormone replacement therapy (HRT) after menopause, elevates the likelihood of ovarian cancer, notably endometrioid carcinoma (28). Conversely, epidemiological studies show that oral contraceptives can reduce the risk of ovarian cancer (29). Therefore, studying the role of steroid hormones in the malignant transformation of EMs is crucial for developing corresponding therapeutic strategies aimed at preventing its malignant progression.

### 2.3 Inflammation and oxidative stress

During menstruation, declining levels of estrogen and progesterone prepare the uterus for the shedding and expulsion of endometrial tissue, marking the onset of a new menstrual cycle. This carefully coordinated process engages the innate immune system, where neutrophils, macrophages, and natural killer cells collaborate to aid in the clearance of apoptotic endometrial tissue (30). Retrograde menstruation can lead to the retention of biologically functional ectopic endometrial fragments within the peritoneal cavity, triggering periodic immune reactions (31). Research indicates heightened levels of inflammatory cytokines, growth factors, neutrophils, and prostaglandins in the peritoneal fluid of EMs patients (32). Elevated levels of Prostaglandin E Receptor 2(PGE2), tumor necrosis factor-α (TNF-α), nerve growth factor (NGF), C-C Motif Chemokine Ligand 5(CCL5), interleukins (IL), Interleukin8(IL-8), and Interleukin 1β(IL-1β)within ectopic lesions can activate sensory nerve endings, thereby contributing to chronic pelvic pain (33–35), which may transmit pain sensations to the central nervous system (36). Therefore, understanding cellular inflammatory responses is crucial for elucidating the pathological processes of EMs, offering insights into potential therapeutic strategies for this condition.

**Figure 2:**
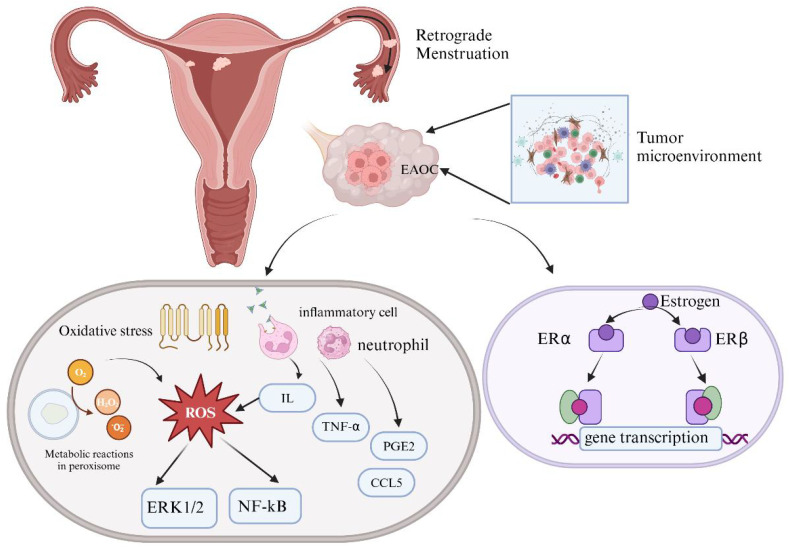
Mechanisms of steroid hormones and oxidative stress in EAOC.

The extracellular matrix cells of ectopic endometrium are often in a microenvironment characterized by iron overload, with high levels of free iron in cyst fluid. Iron can catalyze Fenton reactions generating hydroxyl radicals, which damage DNA and other biomolecules, promoting carcinogenesis (37). Fenton reaction accelerates the production of reactive oxygen species (ROS), oxidative stress resulting from imbalance between excessive ROS and antioxidant defense systems within cells (38). Superoxide anions and hydrogen peroxide (H_2_O_2_) are principal components of ROS, closely linked to inflammation and extracellular matrix degradation. Oxidative stress plays a crucial role in the pathogenesis of EMs (38), damaging normal cell functions, causing DNA damage, stimulating angiogenesis, and thereby promoting EAOC (39). Excessive oxidative stress activates the NF-κB signaling pathway, promoting the production of inflammatory mediators, exacerbating the pathological processes of EMs through Th1 and Th2 immune responses (40). Interleukin10(IL-10) plays a pivotal role in this process by modulating Matrix Metallopeptidase (MMP) activation in serum and peritoneal fluid, ECM remodeling, and angiogenesis. Increased oxidative stress may upregulate oncogenic genes like Cytochrome C Oxidase-2 (COX-2), inhibiting apoptosis and promoting excessive proliferation of ectopic cells (41).Furthermore, oxidative stress enhances the expression of glycoproteins to stimulate vascular endothelial growth factor (VEGF) production, fostering neovascularization and aggravating the disease process (42). Additionally, ROS-activated ERK1/2 protein kinase alters the proliferation and survival of endometrial cells, exhibiting malignant biological behaviors akin to tumor cells (43). In conclusion, the development of EMs is closely associated with oxidative stress, involving multiple cell signaling pathways and regulation of inflammatory mediators, providing a theoretical basis for future therapeutic strategies (**Figure 2**).

Changes in the ovarian microenvironment can induce malignant transformation of EMs. Excessive accumulation of estrogen and the imbalance between estrogen receptor-α (ERα) and estrogen receptor-β(ERβ) can cause DNA toxicity in ectopic endometrial cells. Additionally, oxidative stress plays a crucial role in EAOC formation by inducing DNA damage, abnormal cell signaling, and disrupting antioxidant defenses through excessive intracellular reactive oxygen species (ROS). Elevated levels of inflammatory cytokines, such as interleukin (IL) and tumor necrosis factor-α (TNF-α), along with increased Prostaglandin E Receptor 2(PGE2) at ectopic sites, can activate sensory nerve endings at the lesion site, leading to chronic pelvic pain. (Created with BioRender.com).

### 2.4 Epigenetic changes

Unlike genetic mutations, epigenetics does not alter the DNA sequence of genes but influences gene expression through mechanisms such as DNA methylation, histone modifications, and abnormal expression of microRNAs. Current research indicates that epigenetics plays a crucial role in the onset and progression of ovarian cancer, with its aberrant modifications closely associated with the grading and staging of the disease (44).

#### 2.4.1 DNA methylation

High methylation of gene promoter regions can lead to gene silencing, whereas low methylation promotes gene transcription and protein expression. In studies of EMs malignant transformation, abnormal methylation of CpG islands (CGIs) in many cancer-related genes has been identified, indicating a significant role of epigenetic changes in this process (45). Research has demonstrated high methylation in the promoter region of the E-cadherin gene (46), as well as loss of PTEN homologs on chromosome 10 and p16, which promote the malignant transformation of EMs (47). Additionally, abnormal methylation in the promoter regions of Long Interspersed Elements-1 (LINE-1) and interleukin-1 is also associated with this process (48). Researchers have found that abnormal methylation plays a critical role in the malignant transformation of EMs. Using Methylation CpG Island Amplification Combined with Differential Analysis (MCA-RDA), nine differentially methylated candidate genes associated with the malignant transformation of ovarian EMs were successfully screened, including Ras Association Domain Family Member 2(RASSF2), SPARC (Osteonectin), Cwcv And Kazal Like Domains Proteoglycan 2(SPOCK2), RUNX Family Transcription Factor 3(RUNX3), Glutathione S-Transferase Zeta 1(GSTZ1), Cytochrome P450 Family 2 Subfamily A (CYP2A), Globoside Alpha-1,3-N-Acetylgalactosaminyltransferase 1(GBGT1), NADH: Ubiquinone Oxidoreductase Core Subunit S1(NDUFS1), ADAM Metallopeptidase Domain 22(ADAM22), and Tripartite Motif Containing 36(TRIM36) (49). The DNA methyltransferase (DNMT) family plays a crucial role in mediating DNA methylation. Fang et al. found that estrogen upregulates DNMT1, promoting high methylation of the RUNX3 promoter and playing an important role in the malignant transformation of EMs (50). Thus, abnormal methylation, particularly in promoter regions, is a common epigenetic event in the malignant transformation of EMs, playing a significant role in EMs progression.

#### 2.4.2 Histone modifications

Histones play a crucial role in chromatin structure by influencing the packaging of DNA around nucleosomes and controlling gene expression. Examples of post-translational modifications (PTMs) on histones include acetylation, phosphorylation, methylation, ubiquitination, among others (51). Among these, histone acetylation and methylation are the most extensively studied. Although there is no definitive evidence linking histone modifications to the process of EMs malignant transformation, intriguingly, inhibition of the methyltransferase Enhancer Of Zeste 2 Polycomb Repressive Complex 2 Subunit(EZH2) and the acetyltransferase Histone Deacetylase (HDAC), which regulate histone modifications, can lead to tumor regression in ovarian cancer mouse models with ARID1A mutations (52). Mutations in the ARID1A gene are found in over 50% of OCCCs (53), suggesting a potential role of histone modifications in the malignant transformation of EMs. Studies indicate significant upregulation of histone deacetylases and their encoded proteins in EMs and ovarian cancer (OC) (54, 55). Additionally, histone methyltransferase enhancer of EZH2 can activate methylation of lysine 27 on histone H3 within nucleosomes, promoting cell proliferation and suppressing transcription of tumor suppressor genes in both conditions (56). Thus, these findings imply a potential correlation of histone modifications between EMs and OC, suggesting these modifications may contribute to the progression of epithelial ovarian malignancy.

#### 2.4.3 MicroRNA

miRNA is a class of short, endogenous single-stranded RNA molecules, typically ranging in length from 19 to 25 nucleotides. At the level of the endometrium, miRNA plays a role in regulating the pathophysiological dynamics associated with the menstrual cycle and reproductive disorders such as EMs and recurrent miscarriage (57). These RNA fragments function by inhibiting the synthesis of specific proteins, thereby influencing various biological processes including cell growth, development, differentiation, metabolism, aging, inflammation, and immune responses (58). Despite extensive literature reviews on the importance of miRNA in EMs and its malignant transformation, consensus on research findings remains elusive.

**Figure 3:**
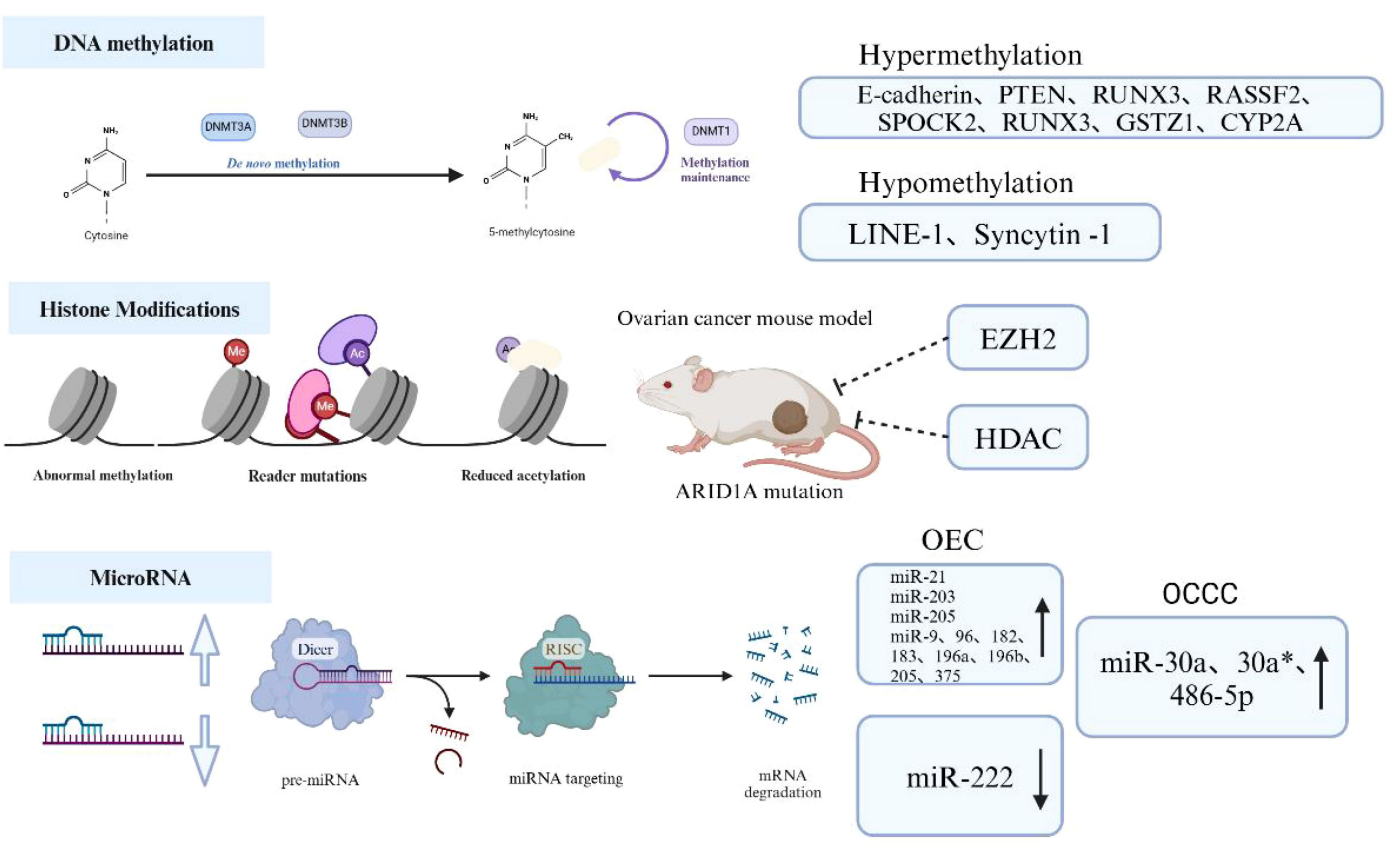
Mechanisms of epigenetic alterations in EAOC.

Common miRNA dysregulations in EMs include the miR-200 family, miR-143, miR-145, miR-20a, and miR-199a. Particularly, dysregulation of the miR-200 family is observed in both EMs and its malignant transformation, suggesting its close association with EMs progression (59). Furthermore, studies have focused on exploring miRNA expression profiles in different tissue types of malignant transformation of EMs. It has been found that miR-21, miR-203, and miR-205 are significantly upregulated in OEC, while miR-222 is downregulated (60). A second-generation sequencing study has also reported specific upregulation of miRNAs such as miR-9, 96, 182, 183, 196a, 196b, 205, and 375 in OEC, and miR-30a, 30a*, and 486-5p in OCCC (61). Additionally, research indicates that oxidative stress, as an important factor inducing malignant transformation of EMs, interacts with miRNAs to affect multiple processes in the development of EAOC (62). Thus, identifying specific miRNAs that promote the malignant transformation of EMs and exploring related molecular pathways may offer new strategies for preventing this progression (**Figure 3**).

Abnormal hypermethylation of tumor suppressor gene promoters, such as E-cadherin and Phosphatase And Tensin Homolog (PTEN), inhibits tumor suppressor gene expression, while hypomethylation of oncogenes like Long Interspersed Elements-1 (LINE-1) promotes oncogene expression and is associated with EAOC development. Inhibition of methyltransferase Repressive Complex 2 Subunit(EZH2)and acetyltransferase Histone Deacetylase(HDAC), which are involved in regulating histone modifications, can lead to tumor regression in AT-Rich Interaction Domain 1A (ARID1A) mutant ovarian cancer mouse models. ARID1A is a commonly mutated gene in EAOC, suggesting that histone modifications may play a role in EAOC. Additionally, microRNAs are crucial post-transcriptional regulators of gene expression; miR-21, miR-203, and miR-205 are significantly upregulated in ovarian epithelial carcinoma (OEC), while miR-222 is downregulated. Conversely, miR-30a, miR-30a*, and miR-486-5p are significantly upregulated in ovarian clear cell carcinoma (OCCC). (Created with BioRender.com).

## 3 Treatment options

Current treatment approaches for EAOC are primarily informed by research on epithelial ovarian cancer (EOC). The standard initial treatment for EAOC includes adjuvant platinum-based chemotherapy following surgery (63). If first-line chemotherapy fails or disease recurs, salvage chemotherapy is administered. However, the prognosis for advanced and recurrent cases is generally poor, and chemotherapy efficacy significantly declines (64). Therefore, the current treatment strategy focuses on enhancing the efficacy of initial treatments, particularly through improved surgical quality and the integration of chemotherapy drugs, targeted therapies, or immunotherapy.

Despite ongoing advancements, clinical outcomes for OCCC remain poorer compared to those for OEC, largely due to its strong resistance to chemotherapy (65). Researchers have long aimed to improve the treatment outcomes for advanced ovarian cancer through intraperitoneal chemotherapy. Despite the completion of several early clinical trials by the Gynecologic Oncology Group (GOG), including GOG-104,GOG-172, and GOG-252, intraperitoneal chemotherapy has not yet been established as a standard first-line treatment (66–68). This is largely because of issues related to trial design, limited statistical evidence, and the need for more investigation into potential adverse effects. Additionally, global clinical trials, such as JGOG-3016, GOG-262, MITO-7, and ICON-8, have adjusted the cycle and frequency of paclitaxel chemotherapy from every three weeks to weekly, or weekly with carboplatin (69–71). These adjustments have their own advantages and disadvantages, and their impact on treatment efficacy needs further clinical trial validation.

Currently, there is no definitive standard for targeted therapy in EAOC. However, targeted therapies may play a crucial role in improving the prognosis of OCCC, particularly in cases resistant to chemotherapy (72). Furthermore, immunotherapy related to EAOC remains a hot research topic.

PIK3CA is a commonly mutated gene in EAOC, which activates the PI3K/AKT/mTOR pathway (73). Inhibitors targeting this pathway, such as PI3K, AKT, and mTORC1 inhibitors, have been evaluated in clinical trials (74), but no standard treatment drug has emerged yet. However, the potential for targeted therapy remains promising. A study involving exome sequencing of 48 OCCC patients indicated that existing molecular targeted drugs may be effective for some patients, highlighting the value of exome sequencing in EAOC research (75).

Additionally, PARP inhibitors have demonstrated efficacy in the treatment of ovarian cancer (76), particularly in populations with homologous recombination deficiencies due to BRCA1/BRCA2 gene mutations (77). Although only 6-8% of EAOC cases have BRCA1/2 mutations (78), PARP inhibitors might be effective in these mutation cases.

In EAOC, the expression of the angiogenesis factor VEGF is significantly elevated compared to EMs (79). Anti-VEGF antibodies, as angiogenesis inhibitors, play a role in ovarian cancer treatment (80).There is no evidence suggesting that anti-VEGF antibodies are more effective for EAOC compared to other histological types, their potential for combination with other drugs may be further explored in future treatments (81).

(Created with BioRender.com).

## 4 Discussion

Despite ongoing interest in the study of EAOC, progress remains slow. The diagnostic criteria for endometriosis-associated malignancy have long adhered to those established by Sampson and Scott (82). However, given the strong pathological heterogeneity of EMs, the probability of detecting residual ectopic endometrial cells decreases with disease severity. Notably, the disordered growth of tumor cells following malignancy and the potential oversight of atypical or ectopic endometrial cells can lead to misdiagnosis as primary ovarian cancer, significantly increasing the likelihood of such errors. Consequently, the foundational research on EAOC is heavily constrained by the availability of clinical samples, unlike other cancers. Present challenges include the unclear pathogenesis of EMs, lack of characteristic clinical symptoms and non-invasive diagnostic methods, and difficulties in predicting the risk of endometriosis malignancy. Therefore, further research into the relevant molecular mechanisms is urgently needed to guide clinical treatment. This review provides a comprehensive overview of the mechanisms underlying EAOC and the latest therapeutic strategies. There have been relatively few high-quality reviews on EAOC in recent years, with some focusing solely on specific mechanisms. This article updates the relevant literature and mechanism diagrams, offering guidance for further research into EAOC.

## 5 Summary

**Figure 4:**
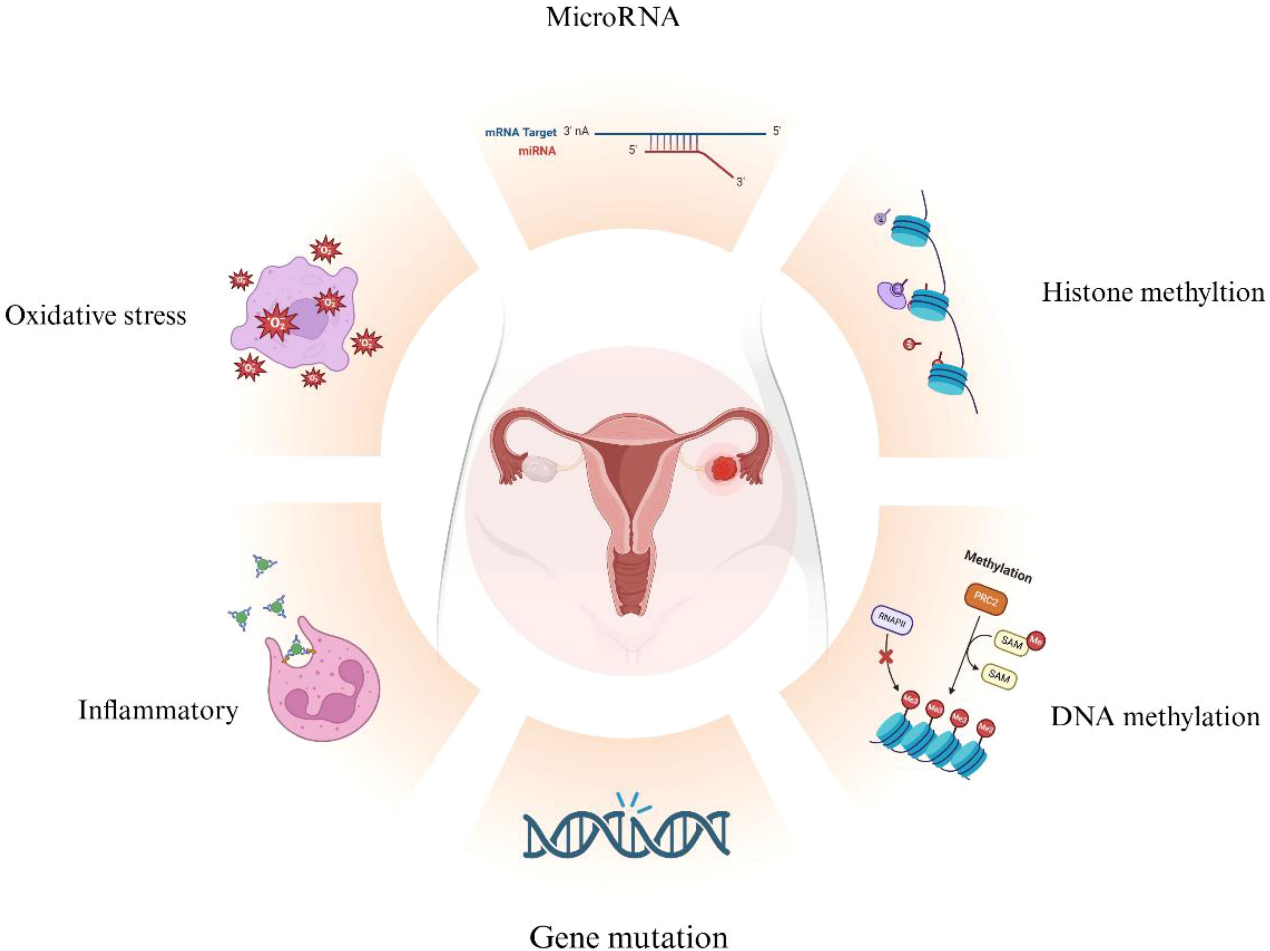
Overview of Mechanisms in EAOC Development. Various mechanisms contribute to the malignant transformation of endometriosis (EMs), including gene mutations, oxidative stress, inflammation, and epigenetic changes related to DNA methylation, histone methylation, and microRNAs. (Created with BioRender.com).

EMs, due to its progressive and invasive growth characteristics, is considered to have potential malignant features. The process of malignant transformation in EMs is complex and may result from various factors acting alone or in combination, including estrogenic effects, immune microenvironment, persistent inflammatory responses, oxidative stress, and genetic mutations (**Figure 4**). For populations of EMs patients at high risk of malignant transformation, the development of early detection methods and close monitoring is crucial to achieve early detection, diagnosis, and treatment, thereby improving patient prognosis and survival outcomes. In-depth analysis of the pathogenesis and malignant transformation mechanisms of EMs can provide new perspectives and strategies for diagnosis and treatment. Future research could explore advanced technologies like next-generation sequencing and whole transcriptome sequencing for personalized diagnostics, aiming to identify driver mutations and candidate genes linked to EMs’ malignant transformation. Such endeavors may enhance the precision of targeted therapies and immunotherapeutic options for EMs patients, underscoring the need for continued investigation.
